# Correction: Cationic pillar[6]arene/ATP host–guest recognition: selectivity, inhibition of ATP hydrolysis, and application in multidrug resistance treatment

**DOI:** 10.1039/d5sc90190a

**Published:** 2025-09-04

**Authors:** Guocan Yu, Jiong Zhou, Jie Shen, Guping Tang, Feihe Huang

**Affiliations:** a State Key Laboratory of Chemical Engineering, Center for Chemistry of High-Performance & Novel Materials, Department of Chemistry, Zhejiang University Hangzhou 310027 P. R. China fhuang@zju.edu.cn +86-571-8795-3189 +86-571-8795-3189; b Department of Chemistry, Institute of Chemical Biology and Pharmaceutical Chemistry, Zhejiang University Hangzhou 310027 P. R. China

## Abstract

Correction for ‘Cationic pillar[6]arene/ATP host–guest recognition: selectivity, inhibition of ATP hydrolysis, and application in multidrug resistance treatment’ by Guocan Yu *et al.*, *Chem. Sci.*, 2016, **7**, 4073–4078, https://doi.org/10.1039/C6SC00531D.

It has come to the authors’ attention that an error requiring correction exists in Fig. 4a. The image was inadvertently duplicated during the final typesetting process, resulting in the unintended repetition of visual data. This occurred as a result of a technical oversight in figure assembly and did not reflect any issue with the underlying experimental data.

The correct figure is displayed below.

**Fig. 4:**
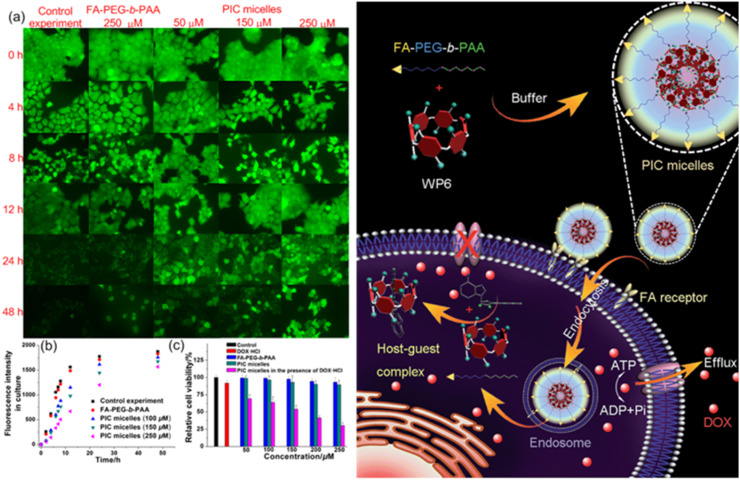
(a) Fluorescence images of the MCF-7/ADR cells stained with calcein-AM incubated without/with **FA-PEG-*b*-PAA** (250 μM), PIC micelles containing different amount of **WP6**. (b) Fluorescence intensity changes of the culture in the presence of **FA-PEG-*b*-PAA** or PIC micelles containing different amounts of **WP6**. (c) Cytotoxicity of DOX·HCl (25 μM), **FA-PEG-*b*-PAA**, PIC micelles, and DOX·HCl (25 μM) loaded PIC micelles with different concentrations of **WP6** against MCF-7/ADR cells. Schematic illustration of the preparation of PIC micelles and possible mechanism to inhibit the efflux pump by forming a host–guest complex **WP6**⊃ATP in the cell.

The Royal Society of Chemistry apologises for these errors and any consequent inconvenience to authors and readers.

